# Inhibitive Effects of Mulberry Leaf-Related Extracts on Cell Adhesion and Inflammatory Response in Human Aortic Endothelial Cells

**DOI:** 10.1155/2013/267217

**Published:** 2013-11-28

**Authors:** P.-Y. Chao, K.-H. Lin, C.-C. Chiu, Y.-Y. Yang, M.-Y. Huang, C.-M. Yang

**Affiliations:** ^1^Department of Nutrition and Health Sciences, Chinese Culture University, Taipei 11114, Taiwan; ^2^Graduate Institute of Biotechnology, Chinese Culture University, Taipei 11114, Taiwan; ^3^Graduate Institute of Applied Life Science, Chinese Culture University, Taipei 11114, Taiwan; ^4^Research Center for Biodiversity, Academia Sinica, Nankang, Taipei 106, Taiwan

## Abstract

Effects of mulberry leaf-related extracts (MLREs) on hydrogen peroxide-induced DNA damage in human lymphocytes and on inflammatory signaling pathways in human aortic endothelial cells (HAECs) were studied. The tested MLREs were rich in flavonols, especially bombyx faces tea (BT) in quercetin and kaempferol. Polyphenols, flavonoids, and anthocyanidin also abounded in BT. The best trolox equivalent antioxidant capacity (TEAC) was generated from the acidic methanolic extracts of BT. Acidic methanolic and water extracts of mulberry leaf tea (MT), mulberry leaf (M), and BT significantly inhibited DNA oxidative damage to lymphocytes based on the comet assay as compared to the H_2_O_2_-treated group. TNF-**α**-induced monocyte-endothelial cell adhesion was significantly suppressed by MLREs. Additionally, nuclear factor kappa B (NF-**κ**B) expression was significantly reduced by BT and MT. Significant reductions were also observed in both NF-**κ**B and activator protein (AP)-1 DNA binding by MLREs. Significant increases in peroxisome proliferator-activated receptor (PPAR) **α** and **γ** DNA binding by MLREs were also detected in M and MT extracts, but no evidence for PPAR **α** DNA binding in 50 **μ**g/mL MT extract was found. Apparently, MLREs can provide distinct cytoprotective mechanisms that may contribute to its putative beneficial effects on suppressing endothelial responses to cytokines during inflammation.

## 1. Introduction

Atherosclerosis is a chronic inflammatory process characterized by increased oxidative stress [1]. The resulting adhesion of monocytes to the vascular endothelium and subsequent migration into the vessel wall are the pivotal early events in atherogenesis [2]. Inflammatory cytokines, tumor necrosis factor-alpha (TNF-*α*), nuclear factor kappa B (NF-*κ*B), and activator protein (AP)-1 are the major redox-sensitive eukaryotic transcription factors that regulate the expression of adhesion molecules [3, 4]. Because the activation of NF-*κ*B and AP-1 can be inhibited to various degrees by different antioxidants, endogenous reactive oxygen species (ROS) may play an important role in these redox-sensitive transcription pathways in atherogenesis [1, 5]. In macrophages, nuclear receptors such as glucocorticoid, estrogen, peroxisome proliferator-activated receptors (PPARs), and liver X receptors negatively modulate inflammatory responses by downregulation of AP-1 and NF-*κ*B [6–8]. PPARs are transcription factors activated by fatty acids and fatty acid-derived eicosanoids [9]. Three isotypes (*α*, *β*/*σ*, and *γ*) expressed in macrophages control the inflammatory status and regulate cholesterol metabolism [10]. A number of phytochemicals commonly used in research have antioxidant activity that can protect cells from ROS-mediated DNA damage that results in mutation and subsequent carcinogenesis [11]. It is evident that quercetin metabolites are distributed in human atherosclerotic lesions [12]. The specific target should therefore be taken into account when evaluating the antioxidant activity of dietary flavonols *in vivo*. Several herbs are consumed to protect against common and serious diseases such as cardiovascular and cerebrovascular events, cancer, and other age-related degenerative diseases as well [13]. These protective effects are considered, in large part, to be related to the various antioxidants contained in them. Several studies have shown that polyphenolic and flavonol substances are the most common compounds in herbs with strong antioxidant activity [14–16]. The bioactive components of herbaceous plants may be responsible for anticancer effects through growth inhibition and apoptosis in human chronic myeloid leukemia K562 cells [17].

*Morus*, a genus of flowering plants in the family Moraceae, comprises 10–16 species of deciduous trees commonly known as mulberries that grow wild and under cultivation in many temperate regions of the world. Mulberries are widespread and important crops for fruit, timber, and silkworm feeding, as well as being excellent amenity trees. *Morus* leaves are also recognized as excellent animal food. Mulberry leaf extracts contain rutin, isoquercetin, and various derivatives of kaempferol and quercetin glycosides that can scavenge 1,1-diphenyl-2-picryl-hydrazyl (DPPH) radical [18] and inhibit the formation of conjugated dienes and thiobarbituric acid-reacting substances (TBARS) by copper-induced oxidative modification of rabbit and human low-density lipoproteins [19]. In addition to antioxidant activity, mulberry leaves and extracts have antimicrobial activity [20], induce apoptosis [18], are antiinflammatory and anti-hyperglycemic, attenuate atherosclerotic lesions in animal models, and lower blood lipids in mild hyperlipidemic patients [21–24]. Although a variety of medicinal herbs including mulberry are known to be potent sources of polyphenols and flavonols, studies on the protective effects of antioxidant activity in mulberry leaf-related extracts (MLREs), especially bombyx faces tea and mulberry leaf tea, on DNA damage and cell adhesion are either scarce or little known. The objectives of this study were to isolate, identify, and evaluate the antioxidant components, antioxidant activity, and extent to which acidic methanolic hydrolysate and water extracts of mulberry leaves can protect DNA in human lymphocytes from oxidative damage induced by H_2_O_2_. The effects of MLREs, including bombyx faces tea (BT), mulberry leaf tea (MT), and mulberry leaf (M) methanolic extracts, on endothelial cell-monocyte adhesion and the inflammatory response were assessed in human aortic endothelial cells (HAEC). Moreover, the effects of MLREs on intracellular redox-sensitive transcriptional pathways such as NF-*κ*B, AP-1, signal transducers, and activators of transcription (STAT)3, and PPAR *α* and *γ*, which may contribute to leukocyte recruitment and vascular inflammation in atherogenesis, were examined by western blot analysis and electrophoretic mobility shift assays. Our study explores the relationship between the composition and content of flavonols and polyphenols having antioxidant efficiency and the prevention of DNA oxidative damage afforded by mulberry leaf-related compounds. The results of our study may help determine the antioxidant activity and anti-inflammatory effects of MLREs.

## 2. Materials and Methods

### 2.1. Mulberry Leaf-Related Compounds Extract Preparation

Mulberry leaf-related compounds (MLRC) were generously provided by the owner of Quan-Ming Silkworm Farm located in Miaoli, Taiwan. Harvested mulberry leaves were divided into eight individual batches and lyophilized by freeze drying in a Freeze Dryer (FD-5060, Panchum Scientific Corp., Taipei, Taiwan), grinding to powder, and storing at −80°C until use. Extracts were produced from 200 mg aliquots of MLRC powder with a 50-fold volume of methanol at 4°C extract overnight and then filtered through Whatman grade no.1 qualitative filter paper. Extracts were concentrated by rotary vacuum evaporator (R205, Buchi, Switzerland), resuspended in 99.5% ethanol to 1 mL, and then stored at −20°C until HAECs treatment.

The edible portion of each MLRC leaf was weighed, lyophilized, ground to powder, and extracted by distilled deionized (dd)H_2_O [25]. The extraction mixture was heated to 90°C in a steam bath and refluxed for 2 h, cooled in the refrigerator, sonicated for 5 min, and diluted to 50 mL with ddH_2_O to form the final extract solution. Individual water extracts were used for the comet assay. Ten mL of 62.5% aqueous methanol containing BHT (2 g/L) was added to 1.25 g lyophilized samples, followed by adding 5 mL 6 M HCl to bring the total volume up to 12.5 mL. The final mixture consisted of 1.2 M HCl in 50% aqueous methanol. Acid hydrolysates methanolic extracts were analyzed for their antioxidant composition, antioxidant activity, and the comet assay.

### 2.2. Polyphenol Assay

Polyphenol content was determined according to the method of Taga et al. [26]. Briefly, standard gallic acid and an aliquot of the acidic methanolic extract were diluted with an ethanol/water (60 : 40, v/v) solution containing 0.3% HCl. Two mL of 2% Na_2_CO_3_ were mixed into each sample of 100 *μ*L and allowed to equilibrate for 2 min before adding 50% Folin-Ciocalteu reagent. Absorbance at 750 nm was measured at room temperature. The standard curve for gallic acid was used to calculate polyphenol levels.

### 2.3. Determination of Total Flavonoids

Total flavonoid contents were determined using the method of Ordoñez et al. [27]. A 0.5 mL 2% AlCl_3_-ethanol solution was added to 0.5 mL of acidic methanolic extract, and absorbance was measured at 420 nm after standing for 1 h at room temperature. Extract samples were evaluated at a final concentration of 0.1 mg/mL. Total flavonoid content was calculated as a quercetin equivalent (mg/g).

### 2.4. Determination of Total Flavonols

Total flavonols in plant extracts were estimated using the method of Kumaran and Joel Karunakaran [28]. Two mL of 2% AlCl_3_ ethanol and 3.0 mL (50 g/L) sodium acetate were added to 2.0 mL acidic methanolic extracts. The absorption at 440 nm was read after 2.5 h at 20°C. Sample extracts were evaluated at a final concentration of 0.1 mg/mL. Total flavonoid content was calculated as a quercetin equivalent (mg/g).

### 2.5. Flavonols Analysis by HPLC

One mL of acid hydrolysates methanolic extract was filtered through 0.45 *μ*m filter prior to 20 *μ*L injection into a high-performance liquid chromatograph (HPLC). Samples were analyzed with a SpectraSystem UV6000LP Photodiode Array Detection System (Thermo Separation Products, San Jose, USA) and an ODS column (250 × 4.6 mm, 5 *μ*m; YMC, ODS-A, YMC Co., Kyoto, Japan). The mobile phase consisted of methanol-water (30 : 70, v/v) with 1% formic acid and 100% methanol. The gradient was 25–74% methanol in 40 min at a flow rate of 0.75 mL/min. the spectrum was recorded at 365 nm for flavonol determination [25].

### 2.6. ABTS/HRP–H_2_O_2_ Assay of Total Antioxidant Activity

The total antioxidant capacity of hydrophilic and lipophilic antioxidants was determined using the horseradish peroxidase catalyzed oxidation of 2,2-azino-bis-(3-ethylbenzothiazoline-6-sulfonic acid) (ABTS) [29]. The reaction mixture contained 0.5 mL of 1000 *μ*M ABTS (in ddH_2_O) and 3.5 mL of 100 *μ*M H_2_O_2_ (in 0.1 M PBS). The reaction was started by the addition of 0.5 mL of 44 U/mL peroxidase (in 0.1 M PBS). After 1 h, 0.05 mL of MLREs was added to the mixture. Absorbance was measured at 730 nm after 5 min. Trolox was used as a standard and the total antioxidant capacity of mulberry extracts was measured as mM Trolox equivalent.

### 2.7. Scavenging Activity on 1,1-Diphenyl-2-picryl-hydrazyl (DPPH) Radical

The scavenging activity on DPPH radical of MLRC acidic methanolic extracts was determined according to the method of Shimada et al. [30]. Briefly, an aliquot of 0.4 mL of acidic methanolic extracts with series dilution was added to 0.8 mL of 1 mM DPPH freshly prepared in methanol, mixed well, and left to stand for 30 min before measuring absorbance at 517 nm. The scavenging effect percentage was calculated as [1 − (OD_517 nm_)/(control OD_517 nm)_] × 100. The IC_50_ of the scavenging effect percentage was then calculated.

### 2.8. Isolated Human Peripheral Blood Lymphocytes

Blood samples were obtained from six donors, four male and two female healthy nonsmokers, that were 24 to 48 years old. Fresh whole blood (20–30 mL) from volunteers was taken with informed consent, and lymphocytes were isolated using a separation solution kit supplemented with Ficoll-Paque Plus lymphocyte isolation sterile solution (Pharmacia Biotech, Sweden) [31]. Cells were harvested within 1 day of blood samples being taken and cultured with AIM V medium containing serum-free lymphocyte medium (Gibco Invitrogen, USA) in a humidified atmosphere of 5% CO_2_ in air at 37°C for 24 h.

### 2.9. Dosage Selected for the Comet Assay

Previously, we demonstrated that the effect of lymphocyte exposure to 10 *μ*M H_2_O_2_ on DNA single-strand break damage was 60-fold greater than the control and with 99.4% cell viability [32]. However, when lymphocytes were exposed to 50 *μ*M H_2_O_2_, the oxidative damage increased significantly compared to the control under 98.2% cell viability. Therefore, 10 *μ*M H_2_O_2_ for the treatment dosage was selected in the current study. Furthermore, when lymphocytes were treated with three concentrations (25, 50, and 100 *μ*g/mL) of acidic methanolic and water extracts from indigenous purple vegetables [33] and herbaceous plants [34], the DNA damage was induced by exposing the lymphocytes to 10 *μ*M H_2_O_2_ and shown the protection effects. Consequently, these concentrations of the extracts were chosen for the effect treatments.

### 2.10. Cell-Viability Testing

After culturing, lymphocytes were exposed to each MLREs. Each lymphocyte solution was used at three concentrations (25, 50, and 100 *μ*g/mL) for 30 min at 37°C. DNA damage was induced by exposing lymphocytes to H_2_O_2_ (10 *μ*M) for 5 min on ice. Treatment on ice minimized the possibility of cellular DNA repair after H_2_O_2_ injury. Cells were centrifuged at 100 g for 10 min, washed, and resuspended in the same medium as the comet assay. All experiments were carried out in triplicate. Cell viability was tested using the tetrazolium/formazan (MTT) assay [35] prior to and after treatment with MLRC extract or H_2_O_2_.

### 2.11. DNA Single Strand Break Damage Estimation Using the Comet Assay

The comet assay was performed as described in Szeto et al. [36] with acidic methanolic hydrolysate and water extracts from tested plants. Cultured lymphocytes (10^5^ cells/mL) were embedded in 75 *μ*L of 1% low-melting-point agarose on a microscope slide (precoated with agarose) at 37°C. The gel was allowed to set at 4°C and cells were lysed for a period of at least 2 h in lysis buffer at 4°C. Cells were then alkaline-unwound, following which electrophoresis was carried out using the electrophoresis buffer at 4°C for 15 min at 25 vDC and 300 mA. All steps were conducted under dim light to prevent additional DNA damage. Following electrophoresis, slides were dipped into a neutralization buffer and stained with ethidium bromide. The comet-like images resulting from the extension of DNA were scored as a reflection of the single strand breaks under a fluorescence microscope (Zeiss-Axiovert 100, Zeiss, Germany). Triplicate slides were prepared for each experimental point sample, and 50 comet-like images selected at random per slide to determine average DNA damage values. A computerized image analysis system (VisCOMET 1.6, Impuls GmbH, Germany) was employed to analyze two comet parameters, DNA damage by tail DNA percentage [(total brightness of tail area/total brightness of total area) × 100%] and tail moment (tail length × tail DNA%). The inhibition percentage of tail DNA% and tail moment were calculated relative to the 10 *μ*M H_2_O_2_ treated group.

### 2.12. Cell Cultures and Treatment

HAECs (Clonetics Corp.) were grown in Medium 200 (GIBCO Invitrogen) supplemented with 1% low serum growth supplement (LSGS; GIBCO Invitrogen) and 10% FBS (GIBCO Invitrogen) in an atmosphere of 95% air and 5% CO_2_ at 37°C in plastic flasks in an incubator (Astec Co.) as described by Vielma et al. [37]. The human monocytic cell line, U937 (American Type Culture Collection) was grown in a suspension culture in RPMI-1640 (GIBCO Invitrogen) containing 10% FBS (Sigma) and 1% antibiotic-antimycotic mixture (Sigma) under an atmosphere of 95% air and 5% CO_2_ at 37°C. After incubation with MLREs and aspirin, or TNF-*α*, cell viability was assessed using the tetrazolium/formazan (MTT) assay [35]. The adhesion assays were performed as described by Zhu and Loft [38]. Briefly, U937 cells were labeled with a fluorescent dye, incubated with 10 *μ*mol/L 2,7-bis (2-carboxyethyl)-5 (6)-carboxyfluorescein acetoxymethyl ester at 37°C for 1 h in RPMI-1640 medium and subsequently washed by centrifugation. Confluent HAECs in 24-well plates were incubated with U937 cells (10^6^ cells/mL) at 37°C for 1 h. Nonadherent leukocytes were removed. The numbers of adherent leukocytes were determined by photographing and counting four randomly chosen fields per well at 100x using a Zeiss Axio Mager Z1 Upright Fluorescence Microscope. Experiments were performed in triplicate and repeated three times. HAECs were incubated with 25 *μ*g and 50 *μ*g of MLREs or 10 *μ*M aspirin for 18 h, followed by treating with 2 ng/mL TNF-*α* for 6 h in the NF-*κ*B p65, AP-1, and STAT-3 expression assays.

### 2.13. Nuclear Protein Isolation

Protein extracts were prepared as described by Min et al. [39]. Briefly, after cell activation for the indicated times, cells were washed in 1 mL ice-cold PBS, centrifuged at 400 g for 5 min, resuspended in 400 *μ*L ice-cold hypotonic buffer (10 mM HEPES, 1.5 mM MgCl_2_, 0.1 mM EDTA, 10 mM KCl, 1 mM DTT, 0.5 mM PMSF, pH 7.9), incubated on ice for 10 min, vortexed, and centrifuged at 15,000 g for 30 sec. The supernatant was collected and stored at −70°C for cytosolic protein analysis. Pelleted nuclei were gently resuspended in 44.5 *μ*L ice-cold extraction buffer (20 mM HEPES, pH 7.9 with 1.5 mM MgCl_2_, 0.42 M NaCl, 0.2 mM EDTA, and 25% glycerol) with 5 *μ*L of 10 mM DTT and 0.5 *μ*L of Protease Inhibitor Cocktail (Sigma), incubated on ice for 20 min, vortexed, and centrifuged at 15,000 g for 5 min at 4°C. Aliquots of the supernatant containing nuclear proteins were frozen in liquid nitrogen and stored at −70°C.

### 2.14. Western Blot Analysis

Nuclear lysates were subjected to 12% SDS-PAGE (Bio-Rad Laboratories), after which proteins were transferred to a PVDF membrane (Bio-Rad). Membranes were probed with a mouse monoclonal NF-*κ*B p65 antibody (BD Biosciences). After incubation in a secondary antibody solution consisting of IRDye 700CW-conjugated goat anti-mouse IgG (LI-COR Biosciences) for 60 min at room temperature with gentle shaking, the membrane was washed four times for 5 min each at room temperature in PBS with 0.1% Tween-20 and while being gently shaken. After a final rinse, the membrane was scanned using AlphaEaseFC image analysis software (Alpha Innotech Corporation) to analyze spot density. The internal control was set at 100% to determine the relative density of protein expression [40].

### 2.15. Electrophoretic Mobility Shift Assay for NF-*κ*B, AP-1, and STAT3

The binding reaction consisted of 1 *μ*L 10× binding buffer (100 mM TRIS, 500 mM NaCl, 10 mM DTT, pH 7.5), 5 *μ*L H_2_O, 2 *μ*L 25 mM DTT/2.5% Tween-20, 1 *μ*L IRDye 700-labeled EMSA oligonucleotides specific for NF-*κ*B (5′-AGT TGA GGG GAC TTT CCC AGG C-3′ and 3′-CGC TTG ATG ACT CAG CCG GAA-5′**)**, AP-1 (5′-CGC TTG ATG ACT CAG CCG GAA-3′ and 3′-GCG AAC TAC TGA GTC GGC CTT-5′), STAT3 **(**5′-GAT CCT TCT GGG AAT TCC TAG ATC-3′ and 3′-CTA GGA AGA CCC TTA AGG ATC TAG-5′) (Sigma), PPAR-*α* (5′-AAA AAC TGG GCC AAA GGT CT-3′), and PPAR-*γ* (5′-TGA AAC TAG GGT AAA GTT CA-3′) (Sigma), 1 *μ*L poly (dI·dC), and 1 *μ*L nuclear extract (as prepared previously). The mixture was incubated at room temperature for 20 min in the dark. After 1x Orange Loading Dye (LI-COR Biosciences) was added, the binding reaction was loaded onto a native 4% polyacrylamide gel and separated by electrophoresis at 90 vDC for 40 min. The gels were scanned using an Odyssey Infrared Imaging System (LI-COR Biosciences) [40].

### 2.16. Statistical Analysis

Data are expressed as the mean ± standard deviation, and statistical significance was analyzed using one-way ANOVA followed by the Tukey's Range Test at the 0.05 significance level. Pearson's linear correlation was also determined. The means of three replicates are reported.

## 3. Result

### 3.1. Antioxidant Composition and Antioxidant Activity

Polyphenols, flavonoids, and flavonols were abundant in BT at levels of 34.65 ± 0.82 mg/g DW gallic acid, 84.83 ± 8.68 mg/g DW quercetin equivalent, and 25.47 ± 3.86 mg/g DW quercetin equivalent, respectively (Table 1). Anthocyanidins were abundant in BT and ML at levels of 143.32 ± 1.81 and 135.82 ± 0.31 units/g DW, respectively.  Table 2 shows that quercetin and kaempferol were both rich in BTat levels of 2853.33 ± 180.37 and 646.67 ± 50.33 *μ*g/g DW, respectively, while the levels of M were 1666.67 ± 189.03 and 426.67 ± 46.19 *μ*g/g DW, respectively. However, no myricetin or morin was detected in the MLRC (Table 2). Thus, these MLREs displayed variations in their antioxidant substances levels.

MLREs showed antioxidant activity, proving their capacity to scavenge the ABTS radical cation. The antioxidant activity in acidic methanolic hydrolysate sample extracts was expressed in trolox equivalent antioxidant capacity (TEAC) (Table 3). BT showed a significantly higher TEAC value (155.14 ± 2.90 *μ*g/g DW) than MT (102.39 ± 0.95 *μ*g/g DW) and M (76.54 ± 1.61 *μ*g/g DW). TEAC values were positively and significantly (*r* = 0.41, *P* = 0.034) correlated with the content of quercetin among MLREs (Table 4). Thus, different quercetin contents displayed various levels of antioxidant activity. However, M had a significantly high efficacy for DPPH radical scavenging activity (IC_50_  188.83 ± 3.61 *μ*g/mL) compared to BT (IC_50_  669.01 ± 7.23 *μ*g/mL) and MT (IC_50_  836.40 ± 7.08 *μ*g/mL) (Table 3). Hence, each tested sample showed significant differences in scavenging of DPPH radical at IC_50_.

### 3.2. Effects of Acidic Methanolic and Water Extracts from MLRC on H_2_O_2_-Induced DNA Damage to Lymphocytes

**Figure 1:**
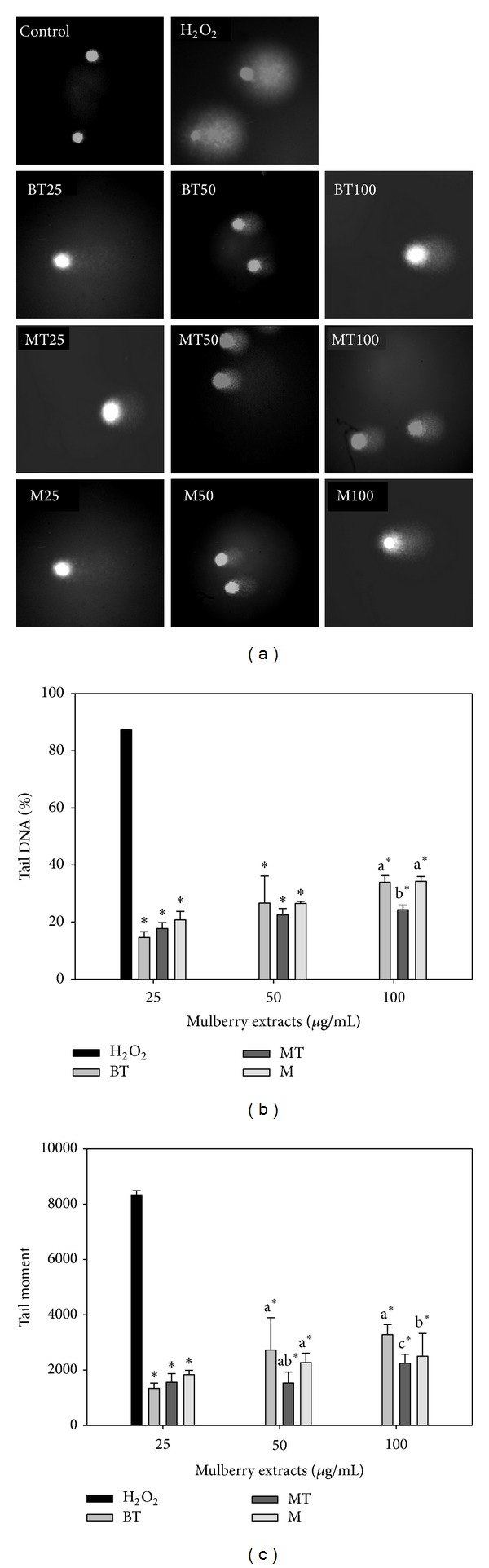
Effects of various acidic methanolic extracts of MLRC on H_2_O_2_-induced DNA damage to lymphocytes. The comet-like images resulting from the extension of DNA (a) were observed using a fluorescence microscope. Tail DNA% (b) and tail movement (c) were exposed to test mulberry leaf-related extracts at 25, 50, and 100 *μ*g/mL and treated with H_2_O_2_ at 10 *μ*M. Values with different letters differ significantly with regard to oxidative damage when comparing different MLRC extracts. *P < 0.05 refers to differences in oxidative damage as compared with 10 *μ*M H_2_O_2_-alone treatment.

The effects of MLRC extracts on cell cytotoxicity were determined by the MTT assay. Lymphocytes were exposed to each of three different MLRC extracts at three concentrations (25, 50, and 100 *μ*g/mL) for 30 min at 37°C. DNA damage was induced by exposing lymphocytes to H_2_O_2_ (10 *μ*M) for 5 min on ice. No mulberry extracts were cytotoxic at the concentrations used, with >98% of cells remaining viable [41]. The comet assay was performed to determine the DNA damaging activity of the plants because it is a sensitive method for monitoring single strand DNA breaks at the single cell level. Any DNA damage that occurred is represented as a tail length (tail migration) of the DNA strand. The effects of pretreatment with acidic methanolic extracts of MLRC on 10 *μ*M H_2_O_2_-induced DNA oxidative damage in human lymphocytes are presented in Figure 1, indicating that there was a protective effect of pretreatment on lymphocytes with each of the MLRC extracts at the lowest dose (25 *μ*g/mL). Lymphocytes pretreated with 25–100 *μ*g/mL extracts of MT extracts experienced a greater level of protection against H_2_O_2_ exposure than lymphocytes exposed to other tested compounds and in a dose-dependent manner. BT had better inhibition efficacy at lower concentration (25 *μ*g/mL), and the maximum protective effect of lymphocyte pretreatment was observed with pretreatment by 25 *μ*g/mL BT, exhibiting 14.62% of tail DNA% compared to treatment only with H_2_O_2_. MT had better inhibition efficacy (84.39%) than M and BT extracts at the 100 *μ*g/mL level (Figure 1(a)). Tested plants showed at least 1342.63 of tail moment in BT extract at the 25 *μ*g/mL level, while the BT extract at 100 *μ*g/mL had the highest tail moment (3281.93) compared to the rest of the acidic methanolic extract samples (Figure 1(b)). Moreover, the trend in tail moment in all samples continued climbing up from 25–100 *μ*g/mL.

**Figure 2:**
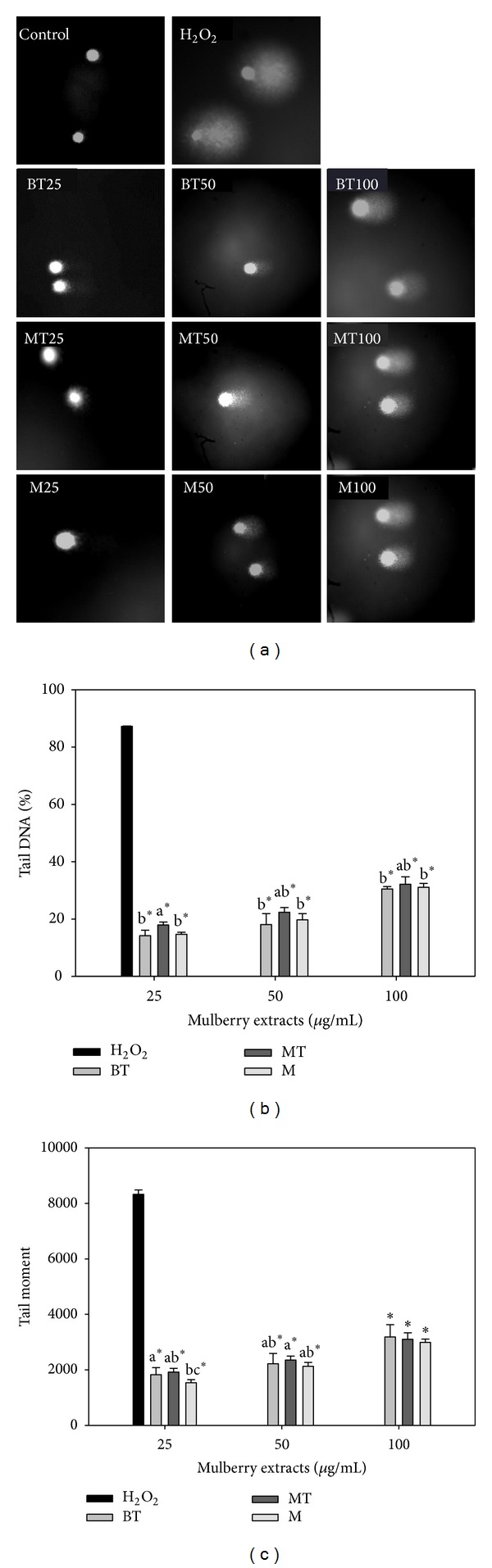
Effects of various water extracts of MLRC on H_2_O_2_-induced DNA damage to lymphocytes. The comet-like images resulting from the extension of DNA (a) were observed using a fluorescence microscope. Tail DNA% (b) and tail movement (c) were exposed to test mulberry leaf-related extractsat 25, 50, and 100 *μ*g/mL and treated with H_2_O_2_ at 10 *μ*M. Values with different letters differ significantly with regard to oxidative damage when comparing different MLRC extracts. *P < 0.05 refers to differences in oxidative damage as compared with 10 *μ*M H_2_O_2_-alone treatment.

The effects of various water extracts of MLRC H_2_O_2_-induced DNA damage to lymphocytes are presented in Figure 2. At lower concentrations, all tested samples had lower tail DNA%, indicating better inhibition efficacies (Figure 2(a)). BT and M had the best inhibition efficacies, with both exhibiting values of 14.22 and 14.69 tail DNA% at 25 *μ*g/mL, respectively. Moreover, MT had a higher tail DNA% than the other extracts at the same dose, and the highest tail DNA% was observed in 32.12% of MT at a concentration of 100 *μ*g/mL. The tail moment of all samples continued to increase up to the 100 *μ*g/mL level (Figure 2(b)). The minimum tail moment occurred in M at 1536.74 in the 25 *μ*g/mL extracts. Moreover, the DNA damage induced by H_2_O_2_ was significantly higher than the DNA damage treated with extracts, and H_2_O_2_ treated had 87.26 in tail DNA% and 8328.84 in tail moment.

### 3.3. Effects of MLREs on Monocyte-Endothelial Cell Adhesion

**Figure 3:**
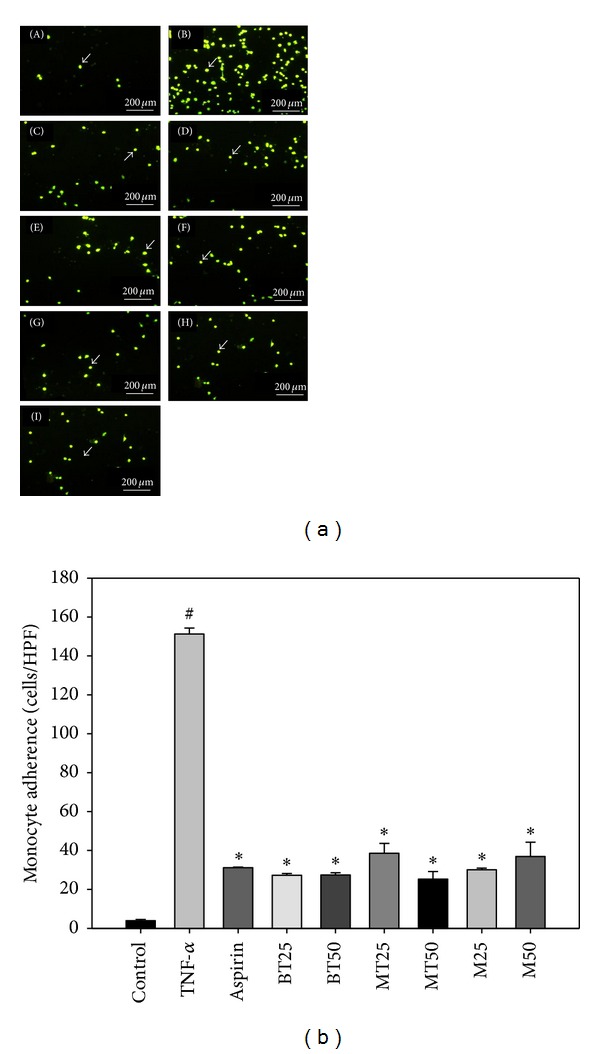
Mulberry leaf related extracts (MLREs) reduce tumor necrosis factor (TNF)-**α**-induced adhesion of monocytes to human aortic endothelial cells (HAECs). (a) The number of adherent U937 cells (marked by arrow) was monitored by fluorescence microscopy under control (a), 2 ng/mL TNF-**α**(b), co-treated with TNF-*a* and aspirin (10 *μ*M) (c), cotreated with TNF-*α* and BT (25 *μ*g/mL) (d), cotreated with TNF-*α* and BT (50 *μ*g/mL) (e), cotreated with TNF-*α* and MT (25 *μ*g/mL) (f), cotreated with TNF-*α* and MT (50 *μ*g/mL) (g), cotreated with TNF-**α**and M (25 *μ*g/mL) (h), and cotreated with TNF-*α* and M (50 *μ*g/mL) (i). (b) HAECs were pretreated with MLREs for 18 h, followed by stimulating with TNF-*α* for 6 h. Fluorescence-labeled monocytic U937 cells were added to HAECs and allowed to adhere for 2 h. Data are expressed as a percentage of TNF-*α*-induced expression (mean ± S.D.) of three experiments. Mean ± S.D. *P < 0.05, BT, MT, and M at 25 and 50 *μ*g/mL versus TNF-*α*; ^#^ *P* < 0.05, control versus TNF-*α*.

As shown in Figure 3, pretreatment of HAECs with 100 *μ*g/mL MLREs for 18 h significantly suppressed the adhesion of U937 monocytes to TNF-*α*-stimulated HAECs similar to that of 10 *μ*M aspirin (positive control). Data also show that 25 and 50 *μ*g/mL BT, MT, and M methanol extracts inhibited the adhesion of U937 monocytes to TNF-*α*-stimulated HAECs by 81.98% and 81.87% (BT), 74.49% and 83.31% (MT), and 80.11% and 75.59% (M), respectively.

### 3.4. Effects of MLREs on NF-*κ*B Activity, and MLREs Inhibits the Binding of NF-*κ*B, AP-1, and STAT3

**Figure 4:**
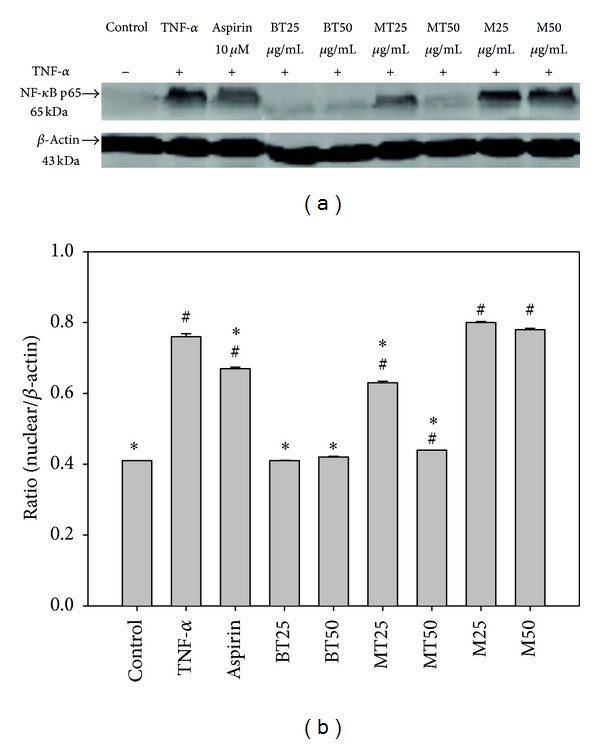
Effects of MLREs on NF-*κ*B activity. After HAECs were pretreated with the indicated samples and incubated with TNF-*α*, nuclear extracts were prepared, and the expression of NF-*κ*B p65 was assessed by western blot analysis. A representative image of three similar results is shown (a). Actin served as the loading control for the nuclear compartment. Semiquantitative analysis of three independent experiments are also shown (b). # indicates a significant difference between the TNF-*α* treated or experimental treatment groups and the control, *P* < 0.05. ∗ indicates a significant difference between the TNF-*α* and experimental treatment groups, BT, MT, and M at 25 and 50 *μ*g/mL.

**Figure 5:**
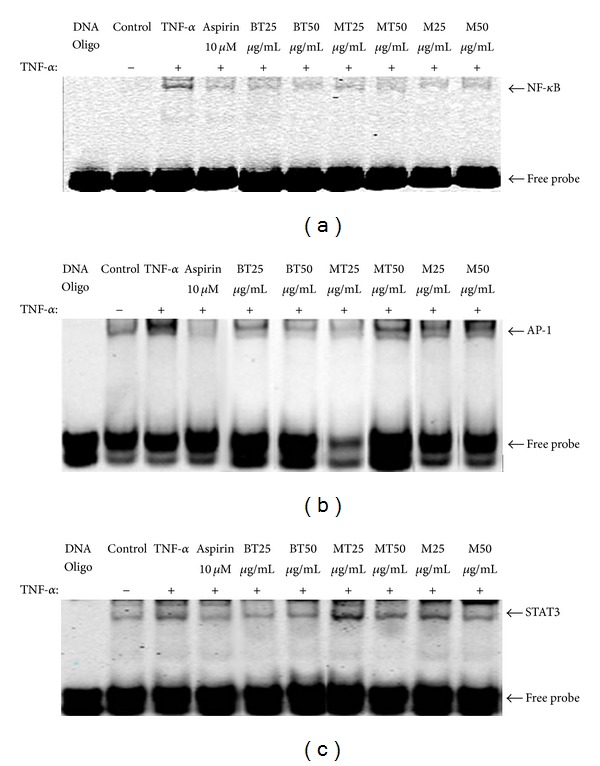
MLREs inhibits the binding of (a) NF-*κ*B, (b) AP-1, and (c) STAT3 to target DNA sequence. NF-*κ*B, AP-1, and STAT3 transcription factor-DNA interactions were assessed by EMSA.

Figure 4 demonstrates that MLREs regulated the DNA-binding activity and expressions of NF-*κ*B in HAECs. In untreated HAECs, NF-*κ*B p65 was only found within the cytosol; however, its nuclear translocation was observed upon treatment with TNF-*α*. Compared to the TNF-*α* group, pretreatment with BT and MT methanol extracts significantly decreased the expression of NF-*κ*B p65 in the nuclear compartment, while the M methanol extract did not reduce the expression of NF-*κ*B p65 activity. Furthermore, an EMSA analysis was carried out to determine if the reduced expression of NF-*κ*B in response to treatment with MLREs resulted in reduced binding of NF-*κ*B to DNA. The treatment of HAECs with TNF-*α* led to increased binding of NF-*κ*B; however, pretreatment of HAECs with BT, MT, and M significantly decreased DNA-bound NF-*κ*B (Figure 5(a)). The increased binding of AP-1 with TNF-*α* resulted from the treatment of HAECs; however, pretreatment of HAECs with BT, MT, and M significantly decreased DNA-bound AP-1 (Figure 5(b)). STAT3 DNA-binding activities to its cognate recognition site were remarkably reduced compared to TNF-*α* treatment (lane 3) in the nuclear fraction, indicating that MLREs at 50 *μ*g/mL inhibited STAT3-binding activities. In addition, the results in Figure 5(c) (lanes 4–10) show that aspirin, MLREs at 50 *μ*g/mL, and BT at 25 *μ*g/mL remarkably decreased STAT3 levels compared to TNF-*α* (lane 3); however, both MT and M at 25 *μ*g/mL increased STAT3-binding activities. These results indicate that only 50 *μ*g/mL MLREs, 25 *μ*g/mL BT, and aspirin block STAT3 activation and might downregulate TNF-*α*-induced expressions of inflammatory signaling pathways.

### 3.5. MLREs Regulated the DNA-Binding Activity and Expressions of PPAR *α* and PPAR *γ* in HAECs

**Figure 6:**
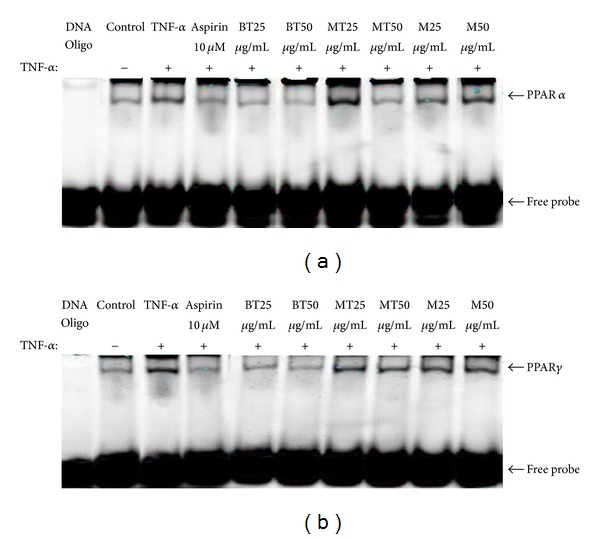
Effect of treatments on the expression of PPAR *α* (a) and PPAR *γ* (b) in TNF-*α*-stimulated HAECs by EMSA.

PPAR *α* and *γ* effectively bound to PPAR *α* and *γ* oligo-IRDye 700 as indicated in lanes 3–10 of Figure 6. Supershifts are bands from the binding of each specific antibody. The binding activities of PPAR *α* and *γ* were detected at baseline. The 25 *μ*g/mL MT extract (lane 7) and 25 and 50 *μ*g/mL M extracts (lanes 9-10) increased activity compared to incubating with TNF-*α* alone (lane 3). However, BT at both 25 and 50 *μ*g/mL extracts showed reduced effects on PPAR *α* and *γ* expression. Moreover, the 50 *μ*g/mL MT extract had no effect on PPAR *α* binding activity but increased the binding activity of PPAR *γ* in comparison to incubation with TNF-*α* alone. These data suggest that MT and M both promoted PPAR *α* and *γ* expression, at least in part, by increasing the DNA binding of transcription factors PPAR *α* and *γ*.

## 4. Discussion

### 4.1. Antioxidant Composition and Antioxidant Activity

BT contains high levels of polyphenols, flavonoids, flavonols, anthocyanidin, quercetin, and kaempferol (Tables 1 and 2). BT also had a higher TEAC value (155.14 mM, Table 3) than the TEAC values of 4.7 mM [42] and 6.4 mM [43] reported for quercetin, as well as 2.42 mM reported for quercetin-3-rutinoside [44]. Furthermore, González-Paramás et al. [45] demonstrated that flavonol contents were significantly and highly correlated (*r* = 0.8) with TEAC values. Kim et al. [46] reported that mulberry leaves contain abundant quercetin and inhibit lipid peroxidation at concentrations of 0.1 and 0.01 mg/mL. In our study, the amount of quercetin was significantly correlated (*r* = 0.41, Table 4) with TEAC value. However, no correlation was found among myricetin, morin, kaempferol, and TEAC, which may be due to the structure required to reinforce the free radical scavenging activity that varies with the type of free radical. Naowaratwattana et al. [18] reported that a 50% methanolic extract of mulberry leaf contained rutin, isoquercetin, and various derivatives of kaempferol and quercetin glycosides, while a water extract contained primarily chlorogenic acid and caffeoylquinic acid derivatives. The IC_50_ of DPPH radical scavenging was 79.4 and 204.2 *μ*g/mL for 50% methanolic and water extracts, respectively [18]. In our study, M extracts contained quercetin and kaempferol (Table 2), which may account for part of the highly scavenged DPPH radical in comparison to the rest of MLREs. Khan et al. [47] demonstrated that the IC_50_ was 220.23 *μ*g/mL, which is much less efficacious than the mulberry leaf acidic methanolic extract IC_50_ of 188.8 *μ*g/mL (Table 3). However, the IC_50_ of *in vitro* free radical scavenging activity of mulberry leaves from Naowaboot et al. [22] was 61.7 ± 2.1 *μ*g/mL, which is more efficacious than the IC_50_ concentration derived in the present study. Taken together, the polyphenol-rich MLREs exhibited distinct cell-free antioxidant activity (i.e., TEAC) as validated by their levels of polyphenols and flavonols, with distinct antioxidant activity strongly accounting for the antioxidant activity of the extracts. The amount of quercetin was especially positively correlated with the observed TEAC values.

### 4.2. Estimation of DNA Single Strand Break Damage from Exposure to Acidic Methanolic and Water Extracts

One of the antioxidant activity tests, scavenging of DPPH radical, was selected due to its quick and simple, but it is a less biologically relevant assay. Previously, we reported that with 2 *μ*g·mL^−1^ of methanolic and water extracts from the indigenous vegetables [33] and herb plants [48] significantly prolonged lag phase of low-density lipoprotein (LDL). The LDL oxidation assay is considered to be one of the most biologically relevant assays [49]. Both studies [33, 48] also showed that the concentration of the tested extracts for scavenging DPPH radical was in higher levels at 10 mg of tested extracts with 2–53% inhibition. Lower concentration was then chosen for the treated dosage in the present study. In addition, lower concentration also could avoid the toxicity effect of higher level of extracts on lymphocytes. Quercetin was found to protect against H_2_O_2_-induced DNA damage in human lymphocytes at 10 *μ*M [50] and at 3.1 to 25 *μ*M [51]. Quercetin was also reported to induce DNA strand breaks in various cell types, but only at higher doses at 100 *μ*M or above [50]. Duthie et al. [50] proposed that the dihydroxy structure of quercetin and myricetin might be essential in protecting DNA against hydrogen peroxide. No such hydroxyl groups are present in the tocopherol molecule. Noroozi et al. [52] demonstrated that, in addition to quercetin, kaempferol could also inhibit H_2_O_2_-induced DNA strand breaks in human lymphocytes. In our study, both BT and M were rich in quercetin and kaempferol (Table 2), which may account for the protective roles of both extracts in preventing DNA strand breaks from human lymphocytes.

### 4.3. MLREs Regulated the DNA-Binding Activity and Expressions of NF-*κ*B, AP-1, STAT3, and PPARs in HAECs

Because MLREs exhibited free radical scavenging, the anti-inflammatory effects of MLREs on TNF-*α*-induced cell adhesion and adhesion molecule expression were determined in the present study. Our results suggest that MLREs and aspirin significantly inhibited TNF-*α*-induced monocyte-endothelial cell adhesion (Figure 3). Significant reductions in NF-*κ*B expression and DNA binding by aspirin, MT extract (25 *μ*g/mL), and BT extract (both 25 and 50 *μ*g/mL) were also observed. The adhesion of monocytes to the vascular endothelium and subsequent migration into the vessel wall are early events in atherogenesis [2]. Our results are consistent with previous research showing that dietary polyphenols such as catechin and quercetin significantly reduce the binding of monocytes to HAECs [53, 54]. The reduced cellular adhesion may be due to the inhibition of cellular adhesion molecule expression, as purple sweet potato leaf extract (PSPLE) reduced both VCAM-1 and ICAM-1 expression [40]. Shibata et al. [55] demonstrated that pretreatment of cultured bovine aortic endothelial cells (BAECs) with mulberry leaf aqueous fractions inhibited TNF-*α*- and LPS-induced expression of Lectin-like Ox-LDL receptor-1 (LOX-1), a cell-surface receptor for atherogenic Ox-LDL (oxidized low-density lipoprotein). It also appears to mediate Ox-LDL-induced inflammation, which may be crucial in atherogenesis. Furthermore, mulberry leaf aqueous fractions inhibited the TNF-*α*-induced activation of NF-*κ*B and phosphorylation of inhibitory factor of NF-*κ*B-alpha (I*κ*B-*α*). Thus, mulberry leaf aqueous fractions suppress TNF-*α*- and LPS-induced LOX-1 gene expression mediated by inhibiting NF-*κ*B activation.

Chan et al. [56] demonstrated that mulberry leaf extract (MLE) was rich in polyphenols. MLE can effectively inhibit the migration of vascular smooth muscle cells (VSMC) by blocking small guanosine triphosphatases (small GTPase) and Akt/NF-*κ*B signals. The Rho family of small GTPases such as Rho, Rac, and Cdc42 acts as a molecular switch to regulate the actin cytoskeleton. The Rho family of GTPases is also reported to be involved in the regulation of NF-*κ*B-dependent transcription [56]. MLE has been shown to possess hypoglycemic effects in an insulin-dependent diabetes mellitus (type 1 diabetes) animal model, and potential hypoglycemic compounds in mulberry leaves were suggested to be 2-*O*-**α**-*D*-galactopyranosyl-DNJ and fagomine [57]. Konno et al. [58] further reported that mulberry latex contains alkaloidal sugar-mimic glycosidase inhibitors with antidiabetic activities such as 1,4-dideoxy-1,4-imino-D-arabinitol, 1-deoxynojirimycin, and 1,4-dideoxy-1,4-imino-D-ribitol. Kim et al. [59] recently showed that treated type 2 diabetes mellitus (T2D) with a 1 : 1 : 1 mixture of ginseng root, mulberry leaf water extract, and banana leaf water extract (6 g/d) for 24 weeks significantly decreased plasma intracellular adhesion molecule-1 (ICAM-1) and vascular cell adhesion molecule-1 (VCAM-1) levels. This indicates suppressed inflammatory responses in T2D but no significant differences in glucose homeostasis control measurement changes. Rigamonti et al. [8] reported that both PPAR *α* and *γ* may be thought of as suppressing the activity of NF*κ*B and AP-1 pathways that reduce inflammatory mediator production in the macrophages of humans and mice. In our study, MT and M promoted PPAR *α* and *γ* expression, at least in part, by increasing DNA binding of transcription factors PPAR *α* and *γ*. PPAR-*α*- and *γ*-dependent signaling pathways may also play important roles in mediating the TNF-*α* receptor signaling pathway by MT and M by suppressing the activity on NF-*κ*B and AP-1 pathways. These pathways may regulate downstream proinflammatory gene expression and adhesion molecular expression. Clinically, PPAR *γ* agonists such as rosiglitazone are widely used in the treatment of T2D [60]. M and MT extracts increased PPAR *γ* activity, which may partly explain why inflammatory responses are suppressed in T2D.

Although reduced NF-*κ*B expression and DNA binding were observed upon treatment with ML, MT, and BT (Figure 5), the mechanism was not assessed and is worthy of further study. In addition, the present results were obtained using *in vitro* studies and may be consistent with an *in vivo* model of atherogenesis similar to that reported by Enkhmaa et al. [21] in which mulberry leaves suppressed the development of atherosclerotic lesions. Previously, we proved that black beans significantly prolong LDL lag time and decrease the atheroma regions of the aortic arch and thoracic aorta in hypercholesterolemic NZW rabbits [61]. We then demonstrated that black soybean extract and PSPLE significantly decreased the adhesion of U937 monocytic cells to TNF-*α*-stimulated HAECs and reduced adhesion molecules expression (VCAM-1 and ICAM-1) as well as NF-*κ*B-p65 expression [37]. This is particularly important given the opposing effects of flavonols from MLREs obtained using *in vitro* and *ex vivo* studies [62].

## 5. Conclusion

Tested MLREs were rich in flavonols and had strong antioxidant activity in protecting lymphocyte DNA from oxidative damage. In particular, BT was rich in quercetin and polyphenols. The antioxidant components of MLREs might downregulate intracellular redox-dependent signaling pathways in HAECs upon TNF-*α* stimulation, which might prevent ROS-mediated endothelial cell dysfunction. MLRCs have anti-inflammatory effects through the modulation of NF-*κ*B, AP-1, STAT3, and PPARs signaling. These chemicals appear to provide distinct cytoprotective mechanisms that might contribute to their putative beneficial effects in suppressing endothelial responses to cytokines during inflammation. These results may benefit further *in vivo* studies to assess the therapeutic potential of phytochemicals as anti-inflammatories for use in cytokine-induced vascular disorders, including atherosclerosis.

## Figures and Tables

**Table 1:** The contents of various antioxidant substances in MLRC.

	Polyphenol mg gallic acid/g DW	Flavonoids mg quercetin equivalent/g DW	Flavonol mg quercetin equivalen/g DW	Athocyanidin unit/g DW
BT	34.65 ± 0.82^a^	84.83 ± 8.68^a^	25.47 ± 3.86^a^	143.32 ± 1.81^a^
MT	23.23 ± 0.54^b^	55.99 ± 10.55^b^	13.26 ± 2.66^b^	88.58 ± 4.12^b^
M	19.87 ± 0.61^c^	49.60 ± 5.41^b^	14.22 ± 1.48^b^	135.82 ± 0.31^a^

All values are means ± S.D. (*n* = 3).

Means within a column with different superscripts (a~c) are significantly different, *P* < 0.05.

**Table 2:** The contents of flavonols in acidic methanolic extracts of MLRC.

Flavonols (*µ*g/g DW)				
Samples	Myricetin	Morin	Quercetin	Kaempferol
BT	ND	ND	2853.33 ± 180.37^a^	646.67 ± 50.33^a^
MT	ND	ND	373.33 ± 23.09^c^	ND
M	ND	ND	1666.67 ± 189.03^b^	426.67 ± 46.19^b^

ND: not detected.

**Table 3:** TEAC values and the IC_50_ of DPPH radical scavenging activity of acidic methanolic hydrolysates of MLRC.

Sample	TEAC (Trolox mM)*	DPPH radical scavenging activity IC_50_ (**μ**g/mL)
BT	155.14 ± 2.90^a^	669.01 ± 7.23^b^
MT	102.39 ± 0.95^b^	836.40 ± 7.08^a^
M	76.54 ± 1.61^c^	188.83 ± 3.61^c^

*TEAC value at sample 100 **μ**g/mL concentration.

**Table 4:** Correlation between antioxidant substances and TEAC values treated with acid hydrolysates of MLRC.

	Flavonols			
	Myricetin	Morin	Quercetin	Kaempferol
TEAC*	—	—	*r* = 0.41 *P* = 0.034	—

—: no significant correlation.

*TEAC values at 100 **μ**g/mL of sample concentration.

## References

[1] Palinski W (2003). United they go: conjunct regulation of aortic antioxidant enzymes during atherogenesis. *Circulation Research*.

[2] Libby P (1995). Molecular bases of the acute coronary syndromes. *Circulation*.

[3] Baeuerle PA, Baltimore D (1996). Nf-*κ*B: ten years after. *Cell*.

[4] Kyriakis JM (1999). Activation of the AP-1 transcription factor by inflammatory cytokines of the TNF family. *Gene Expression*.

[5] Müller JM, Rupec RA, Baeuerle PA (1997). Study of gene regulation by NF-*κ*B and AP-1 in response to reactive oxygen intermediates. *Methods*.

[6] Chinetti G, Fruchart J, Staels B (2003). Peroxisome proliferator-activated receptors: new targets for the pharmacological modulation of macrophage gene expression and function. *Current Opinion in Lipidology*.

[7] Chinetti G, Fruchart JC, Staels B (2006). Transcriptional regulation of macrophage cholesterol trafficking by PPAR*α* and LXR. *Biochemical Society Transactions*.

[8] Rigamonti E, Chinetti-Gbaguidi G, Staels B (2008). Regulation of macrophage functions by PPAR- *α*, PPAR- *γ*, and LXRs in mice and men. *Arteriosclerosis, Thrombosis, and Vascular Biology*.

[9] Bouhlel MA, Brozek J, Derudas B (2009). Unlike PPAR*γ*, PPAR*α* or PPAR*β*/*δ* activation does not promote human monocyte differentiation toward alternative macrophages. *Biochemical and Biophysical Research Communications*.

[10] Chinetti G, Fruchart J-C, Staels B (2003). Peroxisome proliferator-activated receptors and inflammation: from basic science to clinical applications. *International Journal of Obesity*.

[11] Zhu Y, Lin JH-C, Liao H (1998). LDL induces transcription factor activator protein-1 in human endothelial cells. *Arteriosclerosis, Thrombosis, and Vascular Biology*.

[12] Terao J (2009). Dietary flavonoids as antioxidants. *Forum of Nutrition*.

[13] Feng YB, Luo WQ, Zhu SQ (2008). Explore new clinical application of Huanglian and corresponding compound prescriptions from their traditional use. *Zhongguo Zhongyao Zazhi*.

[14] Chu YH, Chang CL, Hsu HF (2000). Flavonoid content of several vegetables and their antioxidant activity. *Journal of the Science of Food and Agriculture*.

[15] Arts IC, van de Putte B, Hollman PC (2000). Catechin contents of foods commonly consumed in The Netherlands. 1. Fruits, vegetables, staple foods, and processed foods. *Journal of Agricultural and Food Chemistry*.

[16] Proteggente AR, Pannala AS, Paganga G (2002). The antioxidant activity of regularly consumed fruit and vegetables reflects their phenolic and vitamin C composition. *Free Radical Research*.

[17] Liu YL, Tang LH, Liang ZQ, You B, Yang S (2010). Growth inhibitory and apoptosis inducing by effects of total flavonoids from Lysimachia clethroides Duby in human chronic myeloid leukemia K562 cells. *Journal of Ethnopharmacology*.

[18] Naowaratwattana W, De-Eknamkul W, De Mejia EG (2010). Phenolic-containing organic extracts of mulberry (*Morus alba* L.) leaves inhibit HepG2 hepatoma cells through G2/M phase arrest and inhibition of topoisomerase II*α* activity. *Journal of Medicinal Food*.

[19] Doi K, Kojima T, Fujimoto Y (2000). Mulberry leaf extract inhibits the oxidative modification of rabbit and human low density lipoprotein. *Biological and Pharmaceutical Bulletin*.

[20] Wang W, Zu Y, Fu Y, Efferth T (2012). *In Vitro* antioxidant and antimicrobial activity of extracts from *Morus alba* L. leaves, stems and fruits. *American Journal of Chinese Medicine*.

[21] Enkhmaa B, Shiwaku K, Katsube T (2005). Mulberry (*Morus alba* L.) leaves and their major flavonol quercetin 3-(6-malonylglucoside) attenuate atherosclerotic lesion development in LDL receptor-deficient mice. *Journal of Nutrition*.

[22] Naowaboot J, Pannangpetch P, Kukongviriyapan V, Kongyingyoes B, Kukongviriyapan U (2009). Antihyperglycemic, antioxidant and antiglycation activities of mulberry leaf extract in streptozotocin-induced chronic diabetic rats. *Plant Foods for Human Nutrition*.

[23] Padilha MM, Vilela FC, Rocha CQ (2010). Antiinflammatory properties of Morus nigra leaves. *Phytotherapy Research*.

[24] Aramwit P, Supasyndh O, Siritienthong T, Bang N (2013). Mulberry leaf reduces oxidation and c-reactive protein level in patients with mild dyslipidemia. *BioMed Research International*.

[25] Justesen U, Knuthsen P, Leth T (1998). Quantitative analysis of flavonols, flavones, and flavanones in fruits, vegetables and beverages by high-performance liquid chromatography with photo-diode array and mass spectrometric detection. *Journal of Chromatography A*.

[26] Taga MS, Miller EE, Pratt DE (1984). Chia seeds as a source of natural lipid antioxidants. *Journal of the American Oil Chemists’ Society*.

[27] Ordoñez AAL, Gomez JD, Vattuone MA, Isla MI (2006). Antioxidant activities of *Sechium edule* (Jacq.) Swartz extracts. *Food Chemistry*.

[28] Kumaran A, Joel Karunakaran R (2007). *In Vitro* antioxidant activities of methanol extracts of five Phyllanthus species from India. *LWT-Food Science and Technology*.

[29] Cao G, Alessio HM, Cutler RG (1993). Oxygen-radical absorbance capacity assay for antioxidants. *Free Radical Biology and Medicine*.

[30] Shimada K, Fujikawa K, Yahara K, Nakamura T (1992). Antioxidative properties of xanthan on the autoxidation of soybean oil in cyclodextrin emulsion. *Journal of Agricultural and Food Chemistry*.

[31] Cole J, Green MHL, James SE, Henderson L, Cole H (1988). A further assessment of factors influencing measurements of thioguanine-resistant mutant frequency in circulating T-lymphocytes. *Mutation Research*.

[32] Hsu C-Y, Yang C-M, Chen C-M, Chao P, Hu S (2005). Effects of chlorophyll-related compounds on hydrogen peroxide induced DNA damage within human lymphocytes. *Journal of Agricultural and Food Chemistry*.

[33] Tang S-C, Lo H-F, Lin K-H (2013). The antioxidant capacity of extracts from Taiwan indigenous purple-leaved vegetables. *Journal of the Taiwan Society for Horticultural Science*.

[34] Lin K-H, Yang Y-Y, Lo H-F Antioxidant activity of herbaceous plant extracts protect against hydrogen peroxide-induced DNA damage in human lymphocytes.

[35] Cory AH, Owen TC, Barltrop JA, Cory JG (1991). Use of an aqueous soluble tetrazolium/formazan assay for cell growth assays in culture. *Cancer Communications*.

[36] Szeto YT, Collins AR, Benzie IFF (2002). Effects of dietary antioxidants on DNA damage in lysed cells using a modified comet assay procedure. *Mutation Research*.

[37] Vielma S, Virella G, Gorod AJ, Lopes-Virella MF (2002). Chlamydophila pneumoniae infection of human aortic endothelial cells induces the expression of FC *γ* receptor II (Fc*γ*RII). *Clinical Immunology*.

[38] Zhu CY, Loft S (2001). Effects of Brussels sprouts extracts on hydrogen peroxide-induced DNA strand breaks in human lymphocytes. *Food and Chemical Toxicology*.

[39] Min YD, Choi C-H, Bark H (2007). Quercetin inhibits expression of inflammatory cytokines through attenuation of NF-*κ*B and p38 MAPK in HMC-1 human mast cell line. *Inflammation Research*.

[40] Chao PY, Huang YP, Hsieh WB (2013). Inhibitive effect of purple sweet potato leaf extract and its components on cell adhesion and inflammatory response in human aortic endothelial cells. *Cell Adhesion & Migration*.

[41] Yang YY (2004). *The antioxidative capacity in herb plant extracts and their protection role in DNA oxidative damage of lymphocyte [M.S. thesis]*.

[42] Rice-Evans CA, Miller NJ, Paganga G (1996). Structure-antioxidant activity relationships of flavonoids and phenolic acids. *Free Radical Biology and Medicine*.

[43] Strube M, Haenen GR, van den Berg H, Bast A (1997). Pitfalls in a method for assessment of total antioxidant capacity. *Free Radical Research*.

[44] Pietta PG (2000). Flavonoids as antioxidants. *Journal of Natural Products*.

[45] González-Paramás AM, Esteban-Ruano S, Santos-Buelga C, De Pascual-Teresa S, Rivas-Gonzalo JC (2004). Flavanol content and antioxidant activity in winery byproducts. *Journal of Agricultural and Food Chemistry*.

[46] Kim SY, Gao JJ, Kang HK (2000). Two flavonoids from the leaves of *Morus alba* induce differentiation of the human promyelocytic leukemia (HL-60) cell line. *Biological and Pharmaceutical Bulletin*.

[47] Khan MA, Rahman AA, Islam S (2013). A comparative study on the antioxidant activity of methanolic extracts from different parts of *Morus alba* L., (Moraceae). *BMC Research*.

[48] Chung A-L, Lo H-F, Lin K-H (2013). Study on the antioxidant activity in herb plant extracts. *Journal of the Taiwan Society For Horticultural Science*.

[49] Prior RL, Wu X, Schaich K (2005). Standardized methods for the determination of antioxidant capacity and phenolics in foods and dietary supplements. *Journal of Agricultural and Food Chemistry*.

[50] Duthie SJ, Collins AR, Duthie GG, Dobson VL (1997). Quercetin and myricetin protect against hydrogen peroxide-induced DNA damage (strand breaks and oxidised pyrimidines) in human lymphocytes. *Mutation Research*.

[51] Liu GA, Zheng RL (2002). Protection against damaged DNA in the single cell by polyphenols. *Pharmazie*.

[52] Noroozi M, Angerson WJ, Lean MEJ (1998). Effects of flavonoids and vitamin C on oxidative DNA damage to human lymphocytes. *American Journal of Clinical Nutrition*.

[53] Koga T, Meydani M (2001). Effect of plasma metabolites of (+)-catechin and quercetin on monocyte adhesion to human aortic endothelial cells. *American Journal of Clinical Nutrition*.

[54] Meng C, Somers P, Hoong L (2004). Discovery of novel phenolic antioxidants as inhibitors of vascular cell adhesion molecule-1 expression for use in chronic inflammatory diseases. *Journal of Medicinal Chemistry*.

[55] Shibata Y, Kume N, Arai H (2007). Mulberry leaf aqueous fractions inhibit TNF-*α*-induced nuclear factor *κ*B (NF-*κ*B) activation and lectin-like oxidized LDL receptor-1 (LOX-1) expression in vascular endothelial cells. *Atherosclerosis*.

[56] Chan KC, Ho HH, Huang CN, Lin M, Chen H, Wang C (2009). Mulberry leaf extract inhibits vascular smooth muscle cell migration involving a block of small GTPase and Akt/NF-kB signals. *Journal of Agricultural and Food Chemistry*.

[57] Chen F, Nakashima N, Kimura I, Kimura M, Asano N, Koya S (1995). Potentiating effects on pilocarpine-induced saliva secretion, by extracts and N-containing sugars derived from mulberry leaves, in streptozocin- diabetic mice. *Biological and Pharmaceutical Bulletin*.

[58] Konno K, Ono H, Nakamura M (2006). Mulberry latex rich in antidiabetic sugar-mimic alkaloids forces dieting on caterpillars. *Proceedings of the National Academy of Sciences of the United States of America*.

[59] Kim HJ, Yoon KH, Kang MJ (2012). A six-month supplementation of mulberry, Korean red ginseng, and banaba decreases biomarkers of systemic low-grade inflammation in subjects with impaired glucose tolerance and type 2 diabetes. *Evidence-based Complementary and Alternative Medicine*.

[60] Nicholson G, Hall GM (2011). Diabetes mellitus: new drugs for a new epidemic. *British Journal of Anaesthesia*.

[61] Chao PY, Chen YL, Lin YC (2013). Effects of black soybean on atherogenic prevention in hypercholesterolemic rabbits and on adhesion molecular expression in cultured HAECs. *Food and Nutrition Sciences*.

[62] Chen C, Li S, Chen C-YO (2011). Constituents in purple sweet potato leaves inhibit *in vitro* angiogenesis with opposite effects *ex vivo*. *Nutrition*.

